# Analytical model for macromolecular partitioning during yeast cell division

**DOI:** 10.1186/s13628-014-0010-6

**Published:** 2014-09-23

**Authors:** Ali Kinkhabwala, Anton Khmelinskii, Michael Knop

**Affiliations:** 1 Abteilung Systemische Zellbiologie, Max-Planck-Institut für molekulare Physiologie, Otto-Hahn-Str. 11, Dortmund 44227, Germany; 2 Zentrum für Molekulare Biologie der Universität Heidelberg (ZMBH) and Deutsches Krebsforschungszentrum (DKFZ), DKFZ-ZMBH-Allianz, Im Neuenheimer Feld 282, Heidelberg 69120, Germany

**Keywords:** Asymmetric cell division, Yeast mitosis, Cellular transport processes, Organelle segregation, Organelle inheritance, Diffusion, Narrow escape, Bud neck, Protein aggregate, Prion, Extrachromosomal rDNA circle, ERC, Nuclear pore complex, NPC

## Abstract

**Background:**

Asymmetric cell division, whereby a parent cell generates two sibling cells with unequal content and thereby distinct fates, is central to cell differentiation, organism development and ageing. Unequal partitioning of the macromolecular content of the parent cell — which includes proteins, DNA, RNA, large proteinaceous assemblies and organelles — can be achieved by both passive (e.g. diffusion, localized retention sites) and active (e.g. motor-driven transport) processes operating in the presence of external polarity cues, internal asymmetries, spontaneous symmetry breaking, or stochastic effects. However, the quantitative contribution of different processes to the partitioning of macromolecular content is difficult to evaluate.

**Results:**

Here we developed an analytical model that allows rapid quantitative assessment of partitioning as a function of various parameters in the budding yeast *Saccharomyces cerevisiae*. This model exposes quantitative degeneracies among the physical parameters that govern macromolecular partitioning, and reveals regions of the solution space where diffusion is sufficient to drive asymmetric partitioning and regions where asymmetric partitioning can only be achieved through additional processes such as motor-driven transport. Application of the model to different macromolecular assemblies suggests that partitioning of protein aggregates and episomes, but not prions, is diffusion-limited in yeast, consistent with previous reports.

**Conclusions:**

In contrast to computationally intensive stochastic simulations of particular scenarios, our analytical model provides an efficient and comprehensive overview of partitioning as a function of global and macromolecule-specific parameters. Identification of quantitative degeneracies among these parameters highlights the importance of their careful measurement for a given macromolecular species in order to understand the dominant processes responsible for its observed partitioning.

## 1 Background

**Figure 1:**
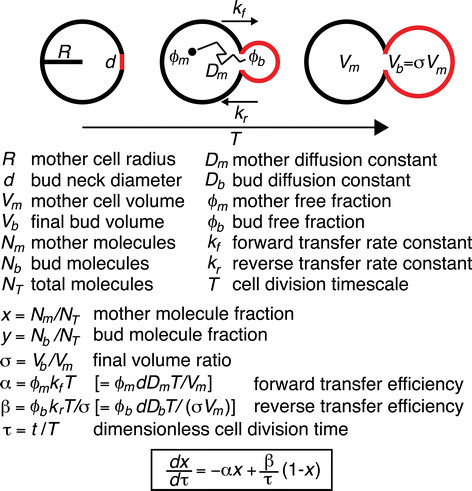
**Model for partitioning of macromolecular content between mother and bud during yeast cell division.** The parameters used in our model are defined. Final partitioning depends only on the two dimensionless parameters *α* = *ϕ*_*m*_*k*_*f*_*T* and *β* = *ϕ*_*b*_*k*_*r*_*T*/*σ*, with additional equalities in brackets corresponding to the specific case of compartmental escape mediated by passive diffusion. All results presented in this manuscript are based on numerical integration of the displayed ordinary differential equation for specific *α* and *β* (Eq. 6).

Reproduction of the yeast *Saccharomyces cerevisiae* by budding is a classical example of asymmetric cell division. The future daughter yeast cell grows as a bud on the surface of the mother cell, receiving macromolecules and organelles that “escape” from the mother compartment through the narrow opening of the bud neck (Figure 1). Movement of macromolecular content from the mother to the bud has been heavily investigated in the last decades. These studies identified various pathways that use motor proteins to transport and regulate the partitioning of organelles such as the nucleus, vacuoles, endoplasmic reticulum, peroxisomes, mitochondria and secretory vesicles (for reviews, see [1]-[3]).

Cells also contain other types of large structures that are close in size to membrane-bound organelles. Some are typically composed of both RNA and proteins and therefore referred to as ribonucleoprotein (RNP) bodies or RNA granules [4]-[6]. Protein granules can be composed of metabolic enzymes, protein degradation machinery or misfolded/damaged proteins in complex with chaperones [7]-[10]. Some of these assemblies exist constitutively, whereas others form in response to changes in the environment. Moreover, the nucleus contains extrachromosomal circular DNA (episomes), of which the extrachromosomal ribosomal DNA circles (ERCs), formed via recombination in the rDNA locus, are a prominent example. Notably, some of these large macromolecular assemblies, namely aggregates of misfolded/damaged proteins and episomes, are largely excluded from the bud during yeast cell division [11]-[13]. The mechanisms responsible for asymmetric partitioning of protein aggregates and episomes are a subject of ongoing discussion.

Asymmetric partitioning of oxidatively damaged protein aggregates is linked to yeast replicative aging [13],[14]. This partitioning appears to involve the actin cytoskeleton [13],[15],[16], possibly even in retrograde movement of aggregates from the bud into the mother [15],[17]. An alternative model, not involving active transport, suggests that slow diffusion of protein aggregates and yeast cell geometry may already largely account for aggregate retention in the mother cell [18]. Additionally, localization of protein aggregates to specialized compartments — the juxtanuclear quality control compartment (JUNQ) and the insoluble protein deposit (IPOD) inclusions — that undergo asymmetric segregation during cell division appears to provide a further contribution to aggregate exclusion from the bud [19],[20].

In early studies, the binding of episomes to structures retained in the mother cell was hypothesized to explain their exclusion from the bud [11], thus contributing to the rejuvenation of the newly born cell [12]. This idea was later revived by a report that nuclear pores — large protein complexes embedded in the nuclear envelope — are retained in the mother compartment during cell division and bind ERCs [21]. However, several studies showed that nuclear pores are not retained in the mother cell [22]-[26] and that tethering to nuclear pores actually promotes segregation of episomes into the bud [27]. Moreover, using live cell tracking of episomes and 3D simulations, Gehlen et al. demonstrated that slow passive diffusion of episomes and the geometry of the yeast nucleus are largely sufficient to explain episome exclusion from the bud [28].

Here we argue that passive diffusion should always be considered when analyzing the partitioning of macromolecular assemblies during cell division. To facilitate the analysis of the role of diffusion, we developed an analytical model of diffusion-driven transfer of macromolecules between the mother and bud compartments of budding yeast. The model is based on a description of diffusive transfer according to the “narrow escape” approximation, which gives the mean time required for a diffusing particle to escape from a compartment through a narrow opening [29]-[32]. Application of this approximation allows conversion of a 3D diffusion problem in a complicated and dynamic geometry to a simple compartmental model for the transfer that is expressible in terms of a single nonlinear ordinary differential equation. The accuracy of this model was confirmed through comparison with the results of previous stochastic simulations [18],[28]. The phase space of all possible solutions of this simple model permits immediate examination of regions for which diffusion is sufficient to drive asymmetric partitioning and those where it is not (therefore implying the presence of other processes like active motor-driven transfer to account for asymmetric partitioning). Finally, important degeneracies among the physical parameters that control the segregation of macromolecular content are revealed by the model.

## 2 Methods

### 2.1 Bidirectional mother-bud transfer assuming a static geometry

According to the “narrow escape” approximation, the escape of particles with diffusion constant *D* from a spherical volume *V* (radius *R*) through a narrow circular opening of diameter *d* can be asymptotically described by an exponential decay with a mean escape time of:

(1) $$t_{e}=K_{e}^{-1}\approx \frac{V}{2\mathrm{dD}}\text{,}$$

where *K*_*e*_ corresponds to the first-order rate constant for escape [29]-[32]. This approximation is exact in the limit of *d* < *R*[29], with the mean escape time accurate to the percent level even for *d* ≈ *R*[31]. In budding yeast, the cytoplasmic content of the mother cell (of diameter ~4–6 μm) does not flow automatically into the bud compartment. Instead, macromolecules and organelles “escape” the mother compartment through the narrow constriction of the bud neck (of diameter ~1 μm), which can be mediated by either passive or active transfer processes. For transfer between the two compartments of the mother and bud, the rate constants for successful transfer between the two compartments are a factor of two lower than the rates of first escape from either compartment due to additional recrossing events at the bud neck [33],[34]. Applying the “narrow escape” approximation in a bidirectional sense to yeast budding leads to the following differential equation governing bidirectional mother-bud transfer via passive diffusion:

(2) $$\frac{dN_{m}}{\mathrm{dt}}=-K_{f}N_{m}+K_{r}N_{b}\text{,}$$

with *N*_*m*_ and *N*_*b*_ the respective particle numbers in mother and bud compartments; *K*_*f*_ = *dD*_*m*_/*V*_*m*_ referring to forward transfer (mother to bud) and *K*_*r*_ = *dD*_*b*_/*V*_*b*_ to reverse transfer; *D*_*m*_ and *D*_*b*_ the compartment-specific diffusion constants (reflecting possible differences in compartmental viscosities); and *d* the bud neck diameter. Total particle conservation, *N*_*T*_ = *N*_*m*_ + *N*_*b*_, implies *dN*_*b*_/*dt* = − *dN*_*m*_/*dt*. Note that the steady state solution (for equal compartment viscosities), *N*_*b*_/*V*_*b*_ = *N*_*m*_/*V*_*m*_, satisfies the homogeneous concentration expected for diffusion in an enclosed geometry in the absence of particle sources or sinks. Rewriting Eq. 2 in terms of concentrations, yields:

(3) $$\frac{dc_{m}}{\mathrm{dt}}=\frac{1}{V_{m}}\frac{dN_{m}}{\mathrm{dt}}=\left(-K_{f}\frac{N_{m}}{V_{m}}+K_{r}\frac{N_{b}}{V_{m}}\right)=-K_{f}c_{m}+K_{f}\frac{D_{b}}{D_{m}}c_{b}\text{.}$$

Note the final equality of the rate constant coefficients for forward and reverse transfer in the case of equal compartmental viscosities, *D*_*m*_ = *D*_*b*_ = *D*. Generalization of Eq. 3 to

(4) $$\frac{dc_{m}}{\mathrm{dt}}=-k_{f}c_{m}+k_{r}c_{b}$$

allows unified treatment of

(I) passive diffusion with equal compartmental viscosities (*D*_*m*_ = *D*_*b*_ = *D*), for which *k*_*r*_ = *k*_*f*_ = *K*_*f*_ = *dD*/*V*_*m*_;

(II) passive diffusion with different compartmental viscosities (*D*_*m*_ ≠ *D*_*b*_), for which *k*_*f*_ = *K*_*f*_ = *dD*_*m*_/*V*_*m*_ and *k*_*r*_ = *K*_*f*_*D*_*b*_/*D*_*m*_ = *dD*_*b*_/*V*_*m*_;

(III) active transport, for which *k*_*f*_ and *k*_*r*_ (as defined with respect to the mother) are determined by directional trafficking processes and can differ in magnitude.

Anomalous diffusion, ubiquitously observed in biological systems [35],[36], can also be considered in our model. In this case, the narrow escape approximation should still apply, requiring only appropriate modification of the relationship given in Eq. 1 between the mean escape time *t*_*e*_ and the now anomalous diffusion constant, with this relationship dependent on the exact mathematical definition of anomalous diffusion.

Transport by convection (bulk flow or cytoplasmic streaming) can also influence macromolecular partitioning [37],[38]. This phenomenon is apparent in large cells such as oocytes or plant cells [39],[40]. However, to our knowledge, the role of bulk flow in yeast cell division has not been considered. In principle, cytoplasmic streaming between mother and bud compartments could result from bud growth during the division process or directed transport of vesicles and organelles into the bud. Our model provides a good testing ground for the contribution of such processes to macromolecular partitioning, especially in cases where diffusive transfer is insufficient to account for the experimentally observed partitioning.

### 2.2 Bidirectional mother-bud transfer with a growing bud

In the above, we have implicitly considered a static mother-bud geometry. Assuming a linear growth of the bud to a final fraction *σ* of the mother volume at the cell division time *T* ($V_{b}\left(t\right)=\sigma \;V_{m}\;\frac{t}{T}$), gives for Eq. 2:

(5) $$\frac{dN_{m}}{\mathrm{dt}}=-k_{f}N_{m}+k_{r}\frac{V_{m}}{V_{b}\left(t\right)}N_{b}=-k_{f}N_{m}+\frac{k_{r}}{\sigma }\frac{T}{t}\left(N_{T}-N_{m}\right)\text{,}$$

where we have implicitly assumed that bud growth is slow compared to the diffusive homogenization timescales within each compartment. Defining *x* = *N*_*m*_/*N*_*T*_, *y* = *N*_*b*_/*N*_*T*_ = 1 − *x*, *τ* = *t*/*T*, *α* = *k*_*f*_*T*, and *β* = *k*_*r*_*T*/*σ* leads to the dimensionless form:

(6) $$\frac{\mathrm{dx}}{\mathrm{d\tau}}=-\alpha \;x+\frac{\beta }{\tau }\left(1-x\right)\text{,}$$

with final partitioning determined solely by the forward transfer efficiency *α* and the reverse transfer efficiency *β*. For passive diffusion, *α* = *dD*_*m*_*T*/*V*_*m*_ and *β* = *dD*_*b*_*T*/(*σ V*_*m*_).

### 2.3 Influence of binding sites on bidirectional mother-bud transfer

Binding sites in the bud (target sites) or in the mother (retention sites) can bias macromolecular partitioning. In the simplest scenario, binding sites are assumed far from saturation with on/off binding fast compared to the transfer timescales. For retention sites, the unbound fraction is then:

(7) $$x_{f}=\frac{1}{1+K_{d}B_{m}}x=\phi_{m}x\text{,}$$

with *B*_*m*_ the binding site concentration and *K*_*d*_ the dissociation constant. Similarly, for target sites, the unbound fraction is:

(8) $$y_{f}=\frac{1}{1+K_{d}B_{b}}y=\phi_{b}y\text{.}$$

Only this free fraction can undergo transfer (either passive or active). Such binding sites are accounted for in our model through inclusion of the free fractions in the forward and reverse transfer efficiencies: *α* = *ϕ*_*m*_*k*_*f*_*T* and *β* = *ϕ*_*b*_*k*_*r*_*T*/*σ*. In addition to binding sites, transient trapping of macromolecules on membranes or within organelles can similarly be accounted for through the reduction in their compartmental free fractions.

### 2.4 Model validation

The accuracy of our model is revealed by comparison to previously published 3D Monte Carlo simulations. For their 3D simulation of passively diffusing protein aggregates, Zhou et al. [18] obtained a bud partitioning of approximately 10% (determined from Figure 7(A) in Zhou et al. [18]), serving as a useful benchmark for our model. Using their parameters (*σ* = 0.61, *R* = 2.5 *μ*m, *d* = 1.25 *μ*m, *D* = 0.001 *μ*m^2^ s^‐ 1^, *T* = 90 min), we obtain 8.4%. However, Zhou et al. [18] did not include bud growth in their simulation. Neglect of bud growth, within the “narrow escape” approximation, requires direct integration of Eq. 2 for a static *K*_*f*_ = *dD*/*V*_*m*_ and *K*_*r*_ = *dD*/*V*_*b*_. This leads to a simple exponential decay of the mother fraction:

(9) $$x\left(t\right)=\frac{V_{m}}{V_{m}+V_{b}}+\frac{V_{b}}{V_{m}+V_{b}}e^{-\left(K_{f}+K_{r}\right)t}\text{.}$$

Using their values, we obtain a final bud fraction of 9.0%, which is in good agreement with their value of approximately 10% (with both fractions far from the maximal value of 38% expected for full volume-based equilibration between the two compartments).

For their 3D simulation of nuclear episomes, which explicitly includes linear growth of the bud nuclear volume and is in all respects identical with the assumptions of our model, Gehlen et al. [28] obtained a bud nucleus partitioning of 25%. Using their parameters (*σ* = 0.64, mother nuclear radius *R* = 0.975 *μ*m, *d* = 0.5 *μ*m, *D* = 0.004 *μ*m^2^ s^‐ 1^, mitosis duration *T* = 9 min), we obtain a 17% bud partitioning, which is in reasonable agreement with the 25% of Gehlen et al. (with both values showing a significant deviation from the maximal result of 39% expected for full volume-based equilibration between the two compartments).

As further confirmation of the underlying assumptions of our model, we note that the intercompartmental gradients at the time of division in the displayed contour maps of both Zhou et al. [18] and Gehlen et al. [28] are confined to a narrow boundary layer at the bud neck with an otherwise flat concentration spanning each individual compartment; this importantly validates the “narrow escape”-based assumption of efficient compartmental homogenization [31].

While these confirmatory results and observations are encouraging, more extensive comparisons of our analytical model with results from 3D simulations are necessary to determine its level of accuracy for different parameters and division geometries. Our model assumes the asymptotically valid “narrow escape” approximation of a uniform exponential distribution for particle transfer between the two compartmental volumes. This allowed us to convert a complicated 3D diffusion problem to a much simpler system of first-order transfer between the two compartments. While the “narrow escape” approximation has been shown to be highly accurate for prediction of the mean escape time even for sizeable escape windows [31], the accuracy of the assumption of a uniform exponential escape (or, in our case, transfer) distribution still remains to be rigorously demonstrated for escape windows of different sizes [41].

### 2.5 Noisy partitioning for low numbers of particles

Reverting from fractions to numbers of molecules in Eq. 6 allows for convenient examination of noise in inheritance:

(10) $$\frac{dN_{m}}{\mathrm{d\tau}}=-\alpha \;N_{m}+\frac{\beta }{\tau }\left(N_{T}-N_{m}\right)\text{.}$$

The dynamics of a single particle that can be present at any given time either in the mother (*Q*_*m*_ = 1,*Q*_*b*_ = 0) or in the bud (*Q*_*m*_ = 0,*Q*_*b*_ = 1) is given by:

(11) $$\frac{dQ_{m}}{\mathrm{d\tau}}=-\alpha \;Q_{m}+\frac{\beta }{\tau }\left(1-Q_{m}\right)\text{.}$$

The summed dynamics of *N*_*T*_ independent particles is therefore:

(12) $$\frac{dQ_{m}^{1}}{\mathrm{d\tau}}+\cdots +\frac{dQ_{m}^{N_{T}}}{\mathrm{d\tau}}=\left[-\alpha \;Q_{m}^{1}+\frac{\beta }{\tau }\left(1-Q_{m}^{1}\right)\right]+\cdots +\left[-\alpha \;Q_{m}^{N_{T}}+\frac{\beta }{\tau }\left(1-Q_{m}^{N_{T}}\right)\right]\text{.}$$

As Eq. 12 is formally equivalent to Eq. 10, consideration of multiple particle dynamics in our model requires only knowledge of the probability of individual particle transfer. Due to this particle independence, the final mother-bud distribution probabilities for *N*_*T*_ particles are given by the corresponding terms in the binomial expansion of the single particle retention/transfer probabilities, ${\left(x+y\right)}^{N_{T}}$. The first term in this series, $x^{N_{T}}$, corresponds to the frequency of null transfer *f* with which all particles are retained in the mother compartment upon completion of cell division. For models in which mother/bud particle concentrations affect transfer rates (particles are not independent), simulations using the Gillespie algorithm [42] would be required to obtain transfer distributions for each *N*_*T*_.

## 3 Results and discussion

Application of the “narrow escape” approximation [29]-[32] in a bidirectional sense to the dividing yeast cell yields a simple compartmental model for diffusive particle transfer between the mother compartment (*m*) and the growing bud (*b*) (Figure 1 and Methods). In this model, the final partitioning of macromolecular content is determined by two dimensionless parameters — the forward (mother to bud) transfer efficiency *α* and the reverse (bud to mother) transfer efficiency *β* — that depend on the forward and reverse transfer rate constants (*k*_*f*_ and *k*_*r*_), the duration of cell division (*T*) and the final bud-to-mother volume ratio (*σ* = *V*_*b*_/*V*_*m*_). The effect of binding to non-partitioned structures (e.g. the cell cortex) is accommodated in the model through the concomitant reduction of free fractions in each compartment (*ϕ*_*m*_ and *ϕ*_*b*_) (Figure 1 and Methods). For passive diffusion, the transfer rate constants are defined, according to the narrow escape approximation, by the compartmental volumes (*V*_*m*_ and *V*_*b*_), the bud neck diameter *d* and the compartment-specific diffusion constants (*D*_*m*_ and *D*_*b*_), yielding for the transfer efficiencies: *α* = *ϕ*_*m*_*dD*_*m*_*T*/*V*_*m*_ and *β* = *ϕ*_*b*_*dD*_*b*_*T*/(*σ V*_*m*_). This formulation achieves a reduction of eight parameters to the two dimensionless transfer efficiencies *α* and *β*, which implies a high level of parameter degeneracy and shows that the same partitioning can be achieved with different parameter combinations. Interestingly, the parameters can be categorized into two groups: parameters that globally influence the partitioning of any molecular content (*d*,*T*,*V*_*m*_,*σ*) and content-specific parameters (*D*_*m*_,*D*_*b*_,*ϕ*_*m*_,*ϕ*_*b*_).

**Figure 2:**
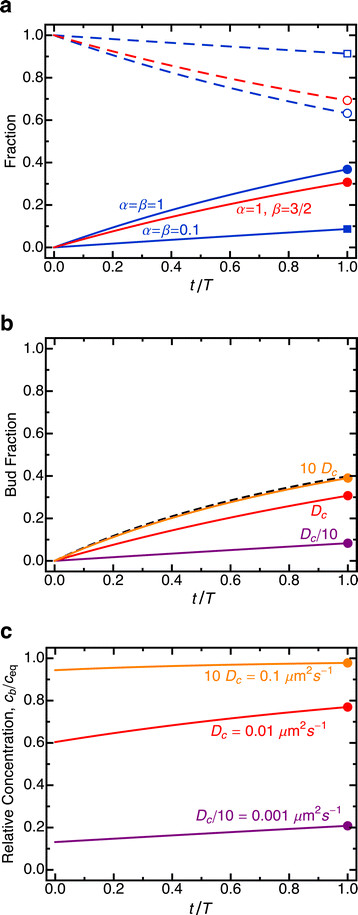
**Partitioning trajectories over the course of cell division. (a)** Partitioning trajectories of the bud (solid) and mother (dashed) for particular values of *α* and *β* (the terminal points are also displayed in Figure 3). The model assumes linear growth of the bud volume over the course of cell division (see Methods). **(b)** Partitioning trajectories for solutions corresponding to different diffusion constants (see Figure 3 as well): *D* = *D*_*c*_ (red, *α* = 1, *β* = 1.5); *D* = 10 *D*_*c*_ (orange, *α* = 10, *β* = 15); *D* = *D*_*c*_/10 (purple, *α* = 0.1, *β* = 0.15). The dashed line gives the asymptotic limit, corresponding to equilibration between mother and bud at all time points. **(c)** Division of the trajectories in **(b)** by the dashed asymptotic trajectory gives the extent of equilibration trajectories. Exact values for the global parameters used to calculate the critical diffusion constant are based on the geometry and division timescale used in Zhou et al. [18] (see Methods).

The model allows calculation of partitioning trajectories over the course of cell division for any given set of parameters, including values of *α* and *β* for which partitioning does not reach equilibrium. Examples of temporal trajectories for different values of *α* and *β* are displayed in Figure 2a. Partitioning equilibration is not achieved for values of *α* and *β* roughly less than 1, defining a critical diffusion constant *D*_*c*_ ≈ *V*_*m*_/(*dT*). Significant retention of molecular species *i* in the mother cell can be expected for *D*_*i*_ < *D*_*c*_ (Figure 2b,c).

**Figure 3:**
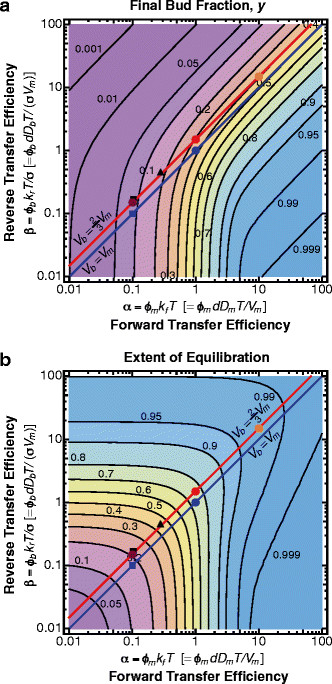
**Partitioning and equilibration of macromolecular content between mother and bud during yeast cell division. (a)** Plot of the solution phase space of the final bud fraction, *y*, as a function of *α* and *β*. The blue line denotes the solution set for passive transfer assuming equal compartmental diffusion constants (*D*_*b*_ = *D*_*m*_), free fractions (*ϕ*_*b*_ = *ϕ*_*m*_), and final volumes (*V*_*b*_ = *V*_*m*_, *σ* = 1). The red line gives the solution set more appropriate for the smaller final bud volume attained in *S. cerevisiae* budding ($V_{b}=\frac{2}{3}V_{m}$, $\sigma =\frac{2}{3}$). Specific symbols in the phase space specify particular solutions for which the corresponding full temporal trajectories have been given in Figure 2a,b. Solutions obtained for the simulation-based parameters of Zhou et al. [18] (■, *y* = 0.084) or for the actual parameters pertaining to the nuclear partitioning of episomes used in Gehlen et al. [28] (▲, *y* = 0.17) are also shown. The solution phase space is color-coded from low (purple) to high (blue) final bud fraction. **(b)** Phase space of the extent of equilibration. For each combination of *α* and *β*, the extent of equilibration was determined as the final bud concentration divided by the fully equilibrated bud concentration that would be obtained in the limit of extremely slow bud growth (see also Figure 2b,c). The solution phase space is color-coded from low (purple) to high (blue) extent of equilibration.

To explore the model, we calculated the bud fraction of macromolecules at the end of the division process *y* = *N*_*b*_/*N*_*T*_ for all possible sets of transfer efficiencies (Figure 3a). Note that the ratio *ρ* = *α*/*β* defines the asymptotic limit $y=\frac{\rho }{1+\rho }$ along each 45° diagonal. The asymptotic limit can be reached by sufficiently slowing down cell division (increasing *T*) such that bidirectional transfer is efficiently equilibrated at each time point. Figure 3b shows the extent of compartmental equilibration, defined as the final bud concentration divided by the fully equilibrated concentration obtained in the asymptotic limit (along the corresponding 45° diagonal), attained at each *α* and *β* in the phase plot.

The blue diagonal in these plots (Figure 3) gives the set of solutions for particles undergoing unbiased passive diffusion in the case of final bud volume equal to the mother volume (corresponding to *y*_eq_ = 0.5 upon complete equilibration). To illustrate the scope of our model, consider the shift from the solution at *α* = 1, *β* = 1 (blue circle) to that at *α* = 0.1, *β* = 0.1 (blue square). This displacement could be achieved in several different ways: a ten-fold increase in the mother volume; or a ten-fold reduction in bud neck diameter, diffusion constant, or division time. Ten-fold reductions of both compartmental free fractions due to efficient retention/target site binding would have the same effect. Our model therefore reveals the exact quantitative degeneracy among these various parameters, obviating much of the need for laborious stochastic simulations of different scenarios (e.g. Zhou et al. [18] and Gehlen et al. [28]). Note that particle enrichment in the bud (*y* > *y*_eq_, corresponding to a higher concentration of particles in the bud) is, of course, not obtainable with unbiased passive diffusion. Solutions above or below the diagonal, the latter including the possibility of bud enrichment, are obtained with different compartmental diffusion constants (*D*_*m*_ ≠ *D*_*b*_), asymmetric distribution of binding sites (leading to *ϕ*_*m*_ ≠ *ϕ*_*b*_) or, more generally, with different transfer rate constants (*k*_*f*_ ≠ *k*_*r*_) for the case of active transport between the compartments.

The offset red diagonal in Figure 3 gives the solution set more appropriate for *S. cerevisiae*, in which the final bud volume is 2/3 the mother volume (here equilibration corresponds to *y*_eq_ = 0.4). Again, only solutions below the red diagonal cover the possibility of particle enrichment in the bud (*y* > *y*_eq_). Due to parameter degeneracy, an increase in the bud neck opening, an extension of the cell division duration, or a corresponding decrease in the cell size will move the partitioning solutions globally along the diagonal towards equilibration. Molecule-specific equilibration can, for example, be achieved by decreasing the number of binding sites in both mother and bud compartments.

For each solution set specified by the final bud-to-mother volume ratio *σ*, fast diffusing particles are partitioned between mother and bud proportional to compartmental volumes, whereas particles with lower diffusion constants are progressively more retained in the mother (Figure 3b). Assembly of rapidly diffusing molecules into larger, less mobile structures could therefore be an important factor in cell fate determination. Misfolded or damaged proteins [10] as well as functional cytosolic content (e.g. metabolic enzymes [7],[8]) can form large supramolecular assemblies. But how slow should diffusion be to limit partitioning in yeast? Assuming typical parameters of *S. cerevisiae* cell division (mother cell radius *R* = 2.5 *μ*m, bud neck diameter *d* = 1.25 *μ*m, and division time *T* = 90 min) [18] yields a critical diffusion constant *D*_*C*_ of 0.01 μm^2^ s^-1^. For comparison, the diffusion constant of the green fluorescent protein GFP is approximately 10 μm^2^ s^-1^ in the yeast cytoplasm [43]. If macromolecular diffusion followed the Stokes-Einstein relation of *D* ∝ *r*^− 1^, where *r* is particle radius, only particles 1000-fold larger than GFP (with radii >3 μm and therefore comparable to or larger than a typical haploid yeast cell) would diffuse slower than 0.01 μm^2^ s^-1^. However, macromolecular crowding within the cytoplasm generally leads to a much stronger dependence of diffusion constant on particle size than predicted by the Stokes-Einstein relation [44]-[46]. In fact, the diffusion constants of two factors associated with yeast replicative aging, protein aggregates marked with the Hsp104 chaperone and extrachromosomal rDNA circles (ERCs), are close to or significantly lower than the critical diffusion constant *D*_*c*_ of 0.01 μm^2^ s^-1^ (0.001 μm^2^ s^-1^ for protein aggregates [18], 0.004 μm^2^ s^-1^ for ERCs [28]), consistent with the hypothesis that passive diffusion may be sufficient to account for their observed asymmetric partitioning [18],[28] (Figure 3, Methods). Precise measurements of cell geometry, compartment-specific diffusion constants, and interactions with subcellular compartments such as the endoplasmic reticulum [47], nucleus and vacuole [20] should help clarify the contribution of slow diffusion relative to mechanisms such as active bud-to-mother transport [17],[18] and binding to retention sites in the mother.

**Figure 4:**
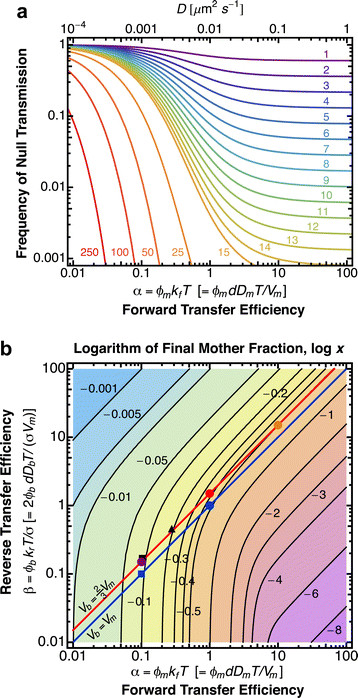
**Examination of noisy partitioning due to low particle numbers. (a)** Frequency of null transfer to the bud (or, equivalently, complete retention in the mother) for different total particle numbers *N*_*T*_ as a function of the forward transfer efficiency *α*, with *β* = 3*α*/2 (i.e., along the red line shown in Figure 3). The upper *x*-axis is expressed in terms of diffusion constant, assuming typical values for yeast budding of *σ* = 2/3, *R* = 2.5 *μ*m, *d* = 1.25 *μ*m, and *T* = 90 min (from Zhou et al. [18]). **(b)** Null transfer frequency phase space. Phase space of the logarithm (base 10) of the final mother fraction, log *x*, corresponding to the bud fraction, *y* = 1 − *x*, displayed in Figure 3a. The frequency of null transfer is $f=x^{N_{T}}$ or log *f* = *N*_*T*_ log *x*. The logarithm of *f* can therefore be efficiently obtained for a given *N*_*T*_ by its product with the displayed log *x* for any value of *α* and *β*. The solution phase space is color-coded from low (purple) to high (blue) logarithm of the final mother fraction.

Prions present another interesting case where diffusion could in principle limit partitioning. These infectious proteins can misfold and aggregate into β-sheet-rich amyloid fibers that catalyze the conversion of newly made proteins into the prion form. Prion propagation by self-templated fiber growth, chaperone-mediated fiber fragmentation and transmission of infectious aggregates to the bud during cell division can generate heritable phenotypes [48],[49] when at least one infectious aggregate is inherited by the daughter cell [50]. Experimental studies indicate that the size of prion aggregates affects their transmission from the mother to the bud, but the mechanisms underlying this size selection remain unclear [51]. According to our model, the probability of the bud receiving no aggregates (frequency of null transmission) depends inversely on the diffusion constant of the aggregates, strongly increasing for diffusion constants below the critical value of 0.01 μm^2^ s^-1^ (Figure 4, Methods). However, measurements with fluorescence correlation spectroscopy indicate that prion aggregates typically diffuse ~50 fold faster than this critical diffusion constant [52]. Thus, diffusion alone appears insufficient to limit aggregate partitioning. Instead, size-dependent interactions of prion aggregates with protein quality control compartments retained in the mother [19],[20] (decreased *ϕ*_*m*_) may explain their exclusion from the bud. Further characterization of aggregate dynamics should help to test this hypothesis and to investigate the alternative possibility of active transport mechanisms.

As diffusion can limit the partitioning of macromolecular content in the context of narrow escape, how does the dividing yeast cell ensure equal partitioning of structures such as large membranous organelles? Decreasing the reverse transfer efficiency (e.g. by increasing the number of binding sites in the bud) can allow enrichment of fast diffusing particles in the bud but has little effect on the partitioning of slow diffusing particles (Figure 3a, solutions below the red diagonal and to the lower left). For the latter, increased forward transfer due to active transport (such that *k*_*f*_ > *k*_*r*_) can obviously lead to bud enrichment. Indeed, most organelles in *S. cerevisiae* are actively transported into the bud by myosin motors acting along actin cables [2],[3]. However, directed transport requires precise monitoring to ensure that some copies of each organelle remain in the mother cell [1]. Although organelle transport appears to be directional (from the mother to the bud), it is noteworthy to consider that directionally is not strictly required. Active transport with frequent random directionality changes would also promote the partitioning equilibration of large organelles, and could be accounted for in our model as an increase in their apparent diffusion constants or, more precisely, as a form of anomalous diffusion (see Methods).

## 4 Conclusions

Our theoretical analysis provides a quantitative overview of the different factors that affect the segregation of cellular content during asymmetric cell division in yeast. In contrast to computationally intensive simulations of particular scenarios, the analytical model derived above provides immediate quantitative assessment of partitioning for arbitrary scenarios defined by their underlying physical parameters. Moreover, our analytical model directly shows how these different physical parameters are related to each other. Analysis of the results of this model suggests that distinct strategies are required to achieve a comparable amount of partitioning of macromolecules and organelles with different mobilities (i.e. different diffusion constants and/or free fractions). Consequently, precise measurements of all parameters are clearly needed to understand how the partitioning of each macromolecular species is accomplished, and to determine whether passive diffusion is sufficient to account for experimentally observed partitioning or whether other processes such as active transport or convection should also be considered. Using fluorescent protein fusions, the mobility of labeled macromolecular species can be investigated with photoconversion, photobleaching or fluorescence correlation spectroscopy, which permit investigation of average mobilities on different time scales, whereas single particle tracking can provide access to the mobilities of individual molecules [53]. Disturbances, including mutant strains, are frequently used to experimentally probe the partitioning process. Since most of the parameters governing partitioning are degenerate, following the partitioning of distinct macromolecular species experimentally upon particular disturbances could be useful for determining whether global parameters or particle-specific parameters are impacted by the disturbance.

## Competing interests

The authors declare that they have no competing interests.

## Authors’ contributions

All authors conceived the research. AKi developed the model with input from AKh and MK. All authors discussed the results and wrote the manuscript. All authors read and approved the final manuscript.
